# Interleukin-19 Mediates Tissue Damage in Murine Ischemic Acute Kidney Injury

**DOI:** 10.1371/journal.pone.0056028

**Published:** 2013-02-26

**Authors:** Yu-Hsiang Hsu, Hsing-Hui Li, Junne-Ming Sung, Wei-Ting Chen, Ya-Chin Hou, Ming-Shi Chang

**Affiliations:** 1 Department of Biochemistry and Molecular Biology, College of Medicine, National Cheng Kung University, Tainan, Taiwan; 2 Department of Planning and Research, National Museum of Marine Biology and Aquarium, Pingtung, Taiwan; 3 Department of Internal Medicine, College of Medicine, National Cheng Kung University, Tainan, Taiwan; 4 Institute of Basic Medical Sciences, College of Medicine, National Cheng Kung University, Tainan, Taiwan; UCL Institute of Child Health, United Kingdom

## Abstract

Inflammation and renal tubular injury are major features of acute kidney injury (AKI). Many cytokines and chemokines are released from injured tubular cells and acts as proinflammatory mediators. However, the role of IL-19 in the pathogenesis of AKI is not defined yet. In bilateral renal ischemia/reperfusion injury (IRI)-induced and HgCl_2_-induced AKI animal models, real-time quantitative (RTQ)-PCR showed that the kidneys, livers, and lungs of AKI mice expressed significantly higher IL-19 and its receptors than did sham control mice. Immunohistochemical staining showed that IL-19 and its receptors were strongly stained in the kidney, liver, and lung tissue of AKI mice. *In vitro*, IL-19 upregulated MCP-1, TGF-β1, and IL-19, and induced mitochondria-dependent apoptosis in murine renal tubular epithelial M-1 cells. IL-19 upregulated TNF-α and IL-10 in cultured HepG2 cells, and it increased IL-1β and TNF-α expression in cultured A549 cells. *In vivo*, after renal IRI or a nephrotoxic dose of HgCl_2_ treatment, IL-20R1-deficient mice (the deficiency blocks IL-19 signaling) showed lower levels of blood urea nitrogen (BUN) in serum and less tubular damage than did wild-type mice. Therefore, we conclude that IL-19 mediates kidney, liver, and lung tissue damage in murine AKI and that blocking IL-19 signaling may provide a potent therapeutic strategy for treating AKI.

## Introduction

Acute kidney injury (AKI), also called acute renal failure (ARF), affects almost 15% of patients admitted to the hospital and results in significant mortality and morbidity [1]. AKI is caused by ischemic and nephrotoxic insults acting alone or in combination. In addition to immediate endothelial and tubular cell injury in the initial phase and persistent vasoconstriction in the early phase of AKI, the subsequent inflammation and recruitment of leukocytes are now recognized as major events leading to the apoptosis and necrosis of endothelial and tubular epithelial cells [2]. Inflammation is characterized by the migration of leukocytes to the activated vascular endothelium via interactions between intercellular adhesion molecules and ligands that enable firm adhesion, followed by transmigration into the interstitium. A number of potent mediators have been identified as generated by the injured epithelial proximal tubular cells that contribute to inflammation, including proinflammatory cytokines such as tumor necrosis factor (TNF), interleukin (IL)-6, IL-1β, IL-8, monocyte chemotactic protein-1 (MCP-1), transforming growth factor-β1 (TGF-β1), and regulated and normal T cell expressed and secreted (RANTES) [3]. Following inflammation, there are two major apoptotic pathways in endothelial and tubular cells. The extrinsic pathway requires the activation of plasma membrane Fas receptor and the activation of caspase 8 by Fas-associated death domain signal transduction, and the intrinsic pathway requires the translocation of Bcl-2-associated X protein (Bax) to the mitochondria, in which Bax forms pores that release cytochrome C and activate caspase 9 [4]. IL-10, an anti-inflammatory cytokine, decreases renal injury in mice treated with either cisplatin or ischemia [5] by inhibiting the inflammatory and apoptotic pathways. These data suggest that manipulating inflammatory cytokines may be a promising strategy for treating AKI; thus, it is important to understand the interactions of various inflammatory cytokines and to identify the key cytokine that is the potential target of therapy, namely, the cytokine that regulates the expression of other proinflammatory cytokines, adhesion molecules, and apoptosis of tubular cells in AKI.

It is known that AKI can have distant effects that potentially alter the function of other organs. Kelly et al [6] demonstrated the effects of renal ischemia on cardiac tissues as shown by induction of IL-1, TNF, and intercellular adhesion molecule 1 mRNA expression as early as 6 h after ischemia. Kramer et al [7] showed that renal ischemic injury led to an increase in pulmonary vascular permeability. Clinical studies [8] have shown that ARF is associated with increased mortality that may be caused by pulmonary complications. Despite these important observations, the pathophysiological mechanism of ischemic ARF complicated by the dysfunction of other organs has not been established.

IL-19 is a cytokine of the IL-10 family (IL-10, -19, -20, -22, -24, and -26) sharing a 20% sequence similarity with IL-10 [9], [10]. Although the functions of IL-19 are not fully understood, it may be a proinflammatory cytokine. Lipopolysaccharide and granulocyte-macrophage colony-stimulating factor increase IL-19 production in mouse monocytes [9]. IL-19 activates STAT3 (signal transducer and activator of transcription-3) through the IL-20 receptor (R) complex (IL-20R1/IL-20R2) [11]. Our recent data [12] demonstrated that IL-19 induces IL-6, TNF-α, and apoptosis in mouse monocytes. IL-19 levels increase in acute systemic inflammatory status, for example, in cardiac surgery patients using a cardiopulmonary bypass [13]; IL-19 is also involved in the pathogenesis of mouse models of septic shock [14] and asthma [15]. Because IL-19 induces cytokines such as TNF-α and IL-6, which are important mediators of AKI, we hypothesized that IL-19 is a mediator of tissue injury in AKI.

## Materials and Methods

### Animal model of renal IRI-induced ARF

All animal experiments were conducted according to the protocols based on the National Institutes of Health standards and guidelines for the care and use of experimental animals. The research procedures were approved by the Animal Ethics Committee of National Cheng Kung University in Taiwan. Eight-week-old male BALB/C mice were anesthetized using pentobarbital (15 mg/kg) and body temperature maintained at 37°C using a homoeothermic blanket. Ischemia was induced by bilateral renal pedicle clamping for 30 minutes. After the clamp was released, the flank was closed, and the mice were placed under a heat lamp. Blood was collected for measurement of BUN. Mice (n = 5) were perfused with phosphate-buffered saline (PBS) to remove the blood from both kidneys and other organs, and then killed 0, 2, 4, and 8 days after surgical procedures. Sham-operated control mice underwent the same surgical procedure, including dissection of the renal pedicle, that except renal clamps were not applied. The kidney tissue isolated from 5 mice in each group was fixed in 3.7% formaldehyde for H&E staining. To analyze severity of AKI in IL-20R1 deficient mice, we used two AKI murine models: renal IRI-induced AKI and HgCl_2_-induced AKI. To evaluate the renal function, serum BUN levels were analyzed. The kidney tissue isolated from the 5 mice in each group was fixed in 3.7% formaldehyde for H&E staining. Tubular damage, defined as “the loss of nuclei”, was estimated by counting the percentage of damaged tubules in 20 high-power fields per section.

### Animal model of HgCl_2_-induced ARF

Eight week-old male Sprague-Dawley rats were subcutaneously (s.c.) injected with HgCl_2_ (3 mg/kg of body weight) in normal saline and then killed 4 days after the injection. The sham controls were untreated rats. Serum BUN level was analyzed to monitor renal function. The left kidneys from three rats in each group were fixed in 3.7% formaldehyde for immunohistochemical staining, and the right kidneys were stored in liquid nitrogen for RNA isolation.

### Histology examination

Tubular damage, defined as “the loss of nuclei”, was estimated by counting the percentage of damaged tubules in 20 high-power fields per section.

### Cell culture

The M-1 cell line was derived from a mouse kidney cortical collecting duct. M-1 cells were purchased from the American Type Culture Collection (ATCC no. CRL-2038; Rockville, MD, USA). The culture medium was a 1∶1 mixture of DMEM and Ham's F12 medium containing 10% FBS. The cells were used between passages 20 and 25. The HepG2 cell line, derived from human hepatoblastomas, was cultured in DMEM medium containing 1.5 g/L glucose and 10% FBS. The A549 cell line was derived from human lung cancer cells, and cultured in DMEM medium containing 4.5 g/L glucose and 10% FBS. All cells were purchased from American Type Culture Collection (Manassas, VA, USA).

### Reagents

Recombinant mouse IL-19, human IL-19, anti-IL-19 monoclonal antibody (mAb) (clone 350105), anti-mouse IL-20R1 mAb (clone 227702), anti-mouse IL-20R2 polyclonal Ab (AF4388) was purchased from R&D Systems Inc. (Minneapolis, MN, USA). SB203580, a p38 MAPK inhibitor, was purchased from Sigma-Aldrich (St. Louis, MO, USA).

### RTQ-PCR

Total RNA was isolated (Invitrogen). Reverse transcription was performed with reverse transcriptase according to the manufacturer's protocol (Clontech). Expression of specific genes (Table S1) was then amplified on a thermocycler (LC 480; Roche Diagnostics) with SYBR Green (Roche Diagnostics) as the interaction agent. The conditions included initial denaturation at 95°C (10 min), followed by 45 cycles at 95°C (15 sec) and 60°C (25 sec). Fluorescent data were acquired during each extension phase. After 45 cycles, a melting curve was generated by slowly increasing (0.1°C/sec) the temperature from 60°C to 95°C, while the fluorescence was measured. Quantification analysis of messenger RNA (mRNA) was normalized with GAPDH, which was used as the housekeeping gene. Relative multiples of changes in mRNA expression were determined by calculating 2^−ΔΔCt^.

### Immunohistochemical staining

Anti-IL-19 mAb, anti-IL-20R1 mAb, and anti-IL-20R2 polyclonal Ab were used for IHC staining according to the manufacturer's instructions. Isotype IgG_1_ was a negative control. Immunoreactivity was detected using a 3-amino-9-ethylcarbazole (AEC) substrate kit for peroxidase (DakoCytomation (formerly Dako), Carpinteria, CA, USA) and nuclei were counterstained with hematoxylin.

### Western blotting

M-1 cells were seeded at 5×10^6^ cells/ml in 10-cm dishes and stimulated with mIL-19 for 24 h. Western blotting with anti-caspase 3, 8, and 9 (Cell Signaling Technology, Danvers, MA, USA) antibodies was done according to the manufacturer's instructions. In addition, the M-1 cells were seeded at 1×10^6^ cells/ml in 6-cm dishes and starved for 21 h. M-1 cells were then stimulated with 5.6 nM (100 ng/ml) of IL-19 for the indicated times. Western blotting with antibodies specific for β-actin, phosphorylated AKT, STAT3, p38 MAPK, JNK, and ERK 1/2 (Cell Signaling Technology) was done according to the manufacturer's instructions.

### Detecting cell death

M-1 cells were seeded at 5×10^5^ cells/ml in 6-well plates and stimulated with different reagents for 24 h. After the culture medium had been removed, the cells were trypsinized and fixed with 50% ethanol. Before flow cytometric analysis, the cells were washed three times with cold phosphate buffer saline and stained with propidium iodide (PI) for 15 min. The presence of the sub-G_0_/G_1_ phase was used as an indicator of cell death. The percentage of cells in the sub-G_0_/G_1_ phase was quantitated using WinMDI 2.8 software.

### Measuring apoptosis

Apoptosis was measured using an annexin-V/PI staining kit (BD Biosciences, San Jose CA, USA). In brief, the cells were treated with different reagents for the indicated times and then trypsinized and resuspended in 50 µl of binding buffer containing fluorescein isothiocyanate (FITC)-conjugated annexin-V and PI. After 15 min, 200 µl of binding buffer was added and the cells were analyzed with a flow cytometer. Ten thousand events were recorded from each assay. Annexin-V-positive cells were considered apoptotic cells.

### ELISA assay

To quantify the protein level of IL-19, mouse kidneys, livers, and lungs in PBS solution were homogenized, centrifuged, and analyzed using direct ELISA. To analyze MCP-1 and TGF-β1 levels in IL-19-treated M1 cells, M-1 cells were seeded and starved for 10 h, and then stimulated with 11.3 nM (200 ng/ml) of IL-19 for 96 h. MCP-1 and TGF-β1 were measured using specific ELISA kits (R&D Systems, Minneapolis, MN). To measure the expression levels of TNF-α, mouse livers and lungs in PBS solution were homogenized, centrifuged, and analyzed using specific ELISA kits (PeproTech).

### Statistical analysis

Prism 5.0 (GraphPad Software) was used for the statistical analysis. A one-way ANOVA nonparametric test (Kruskal-Wallis test) was used to compare the data between groups. Post hoc comparisons were done using Dunn's multiple comparison test. Results are expressed means ± standard deviation (SD). *P*-values <0.05 were considered significant.

## Results

### Higher expression of IL-19 in the kidneys of mice with ischemia/reperfusion injury (IRI)

**Figure 1:**
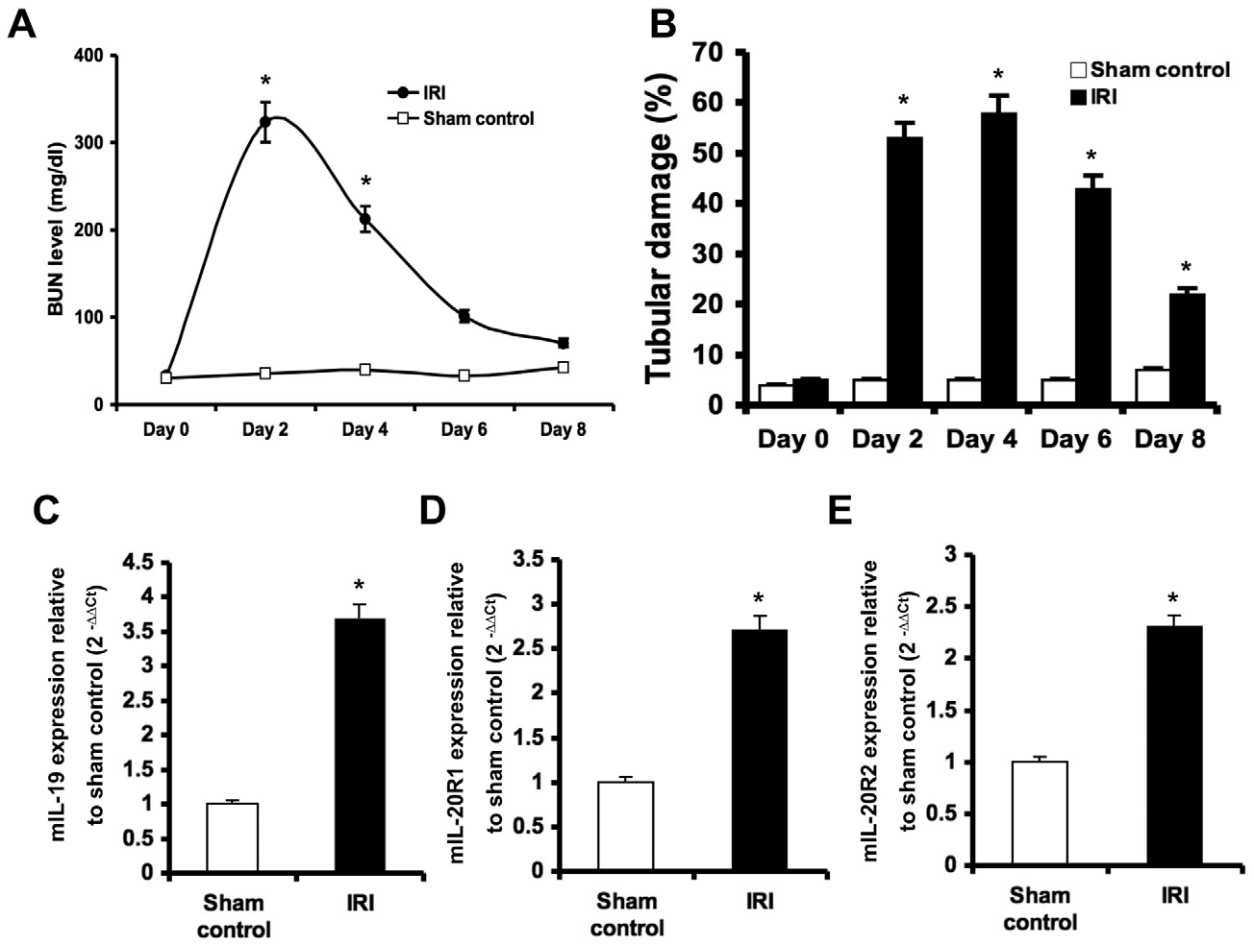
Higher expression of IL-19 and its receptors in ischemic AKI mice. (A) Serum BUN levels and (B) Renal tubular damage were analyzed at the indicated times for monitor renal function in mice with IRI-induced AKI. *P<0.05 compared with the corresponding sham-operated mice. (C–E) Transcript levels of IL-19 and its receptors in the kidney tissue of ischemic AKI mice. Experimental mice were killed 2 days after renal IRI. RTQ-PCR was done with primers specific for IL-19, IL-20R1, and IL-20R2. The relative quantification of PCR products was expressed as 2^−ΔΔCt^, corrected using GAPDH expression, and relative to levels of untreated cells. Data are the means ± SD of three experiments. *P<0.05 compared with sham-operated mice.

To explore the role of IL-19 in ARF, we established an AKI mouse model using IRI. Serum BUN levels and renal tubular damage in IRI mice was significantly higher than in sham control mice (Figure 1A, B), which confirmed the IRI model. Experimental mice were killed two days after IRI had been induced, and RTQ-PCR showed that transcripts of IL-19 and its receptors were higher in the kidney tissue of IRI mice than in sham control mice (Figure 1C–E). In a group of experimental rats with HgCl_2_-induced AKI, IL-19 and its receptors were also upregulated in the injured kidneys (data not shown), which showed the same pattern as those in the IRI mice model. These results suggested that IL-19 is involved in the pathogenesis of AKI *in vivo*.

### The expression of IL-19 and its receptors was higher in the kidneys, lungs, and liver of mice with IRI and mercury chloride (HgCl_2_)-induced AKI rats

**Figure 2:**
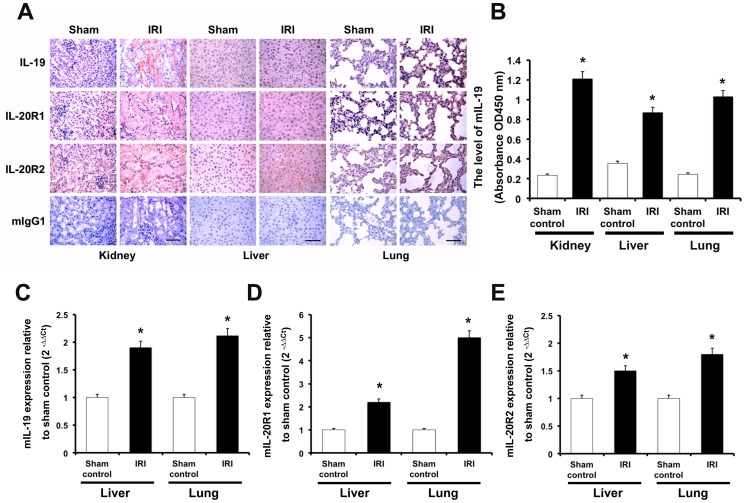
Expression of IL-19 and its receptors in three vital organs of ischemic AKI mice. (A) AKI mice (*n* = 5 in each group) were killed 48 h after renal IRI. Paraffined sections of kidney, liver, and lung tissue were stained using anti-IL-19 mAb, anti-IL-20R1 mAb, and anti-IL-20R2 polyclonal Ab. Anti-mIgG_1_ was a negative control. The reaction was detected using AEC chromogen stain (red), and the nuclei were counterstained with hematoxylin (blue). The bars represent 50 µm. All five mice in each group showed similar patterns. Shown sections are representative of five individual mice. (B) Mouse tissues of each group (*n* = 5) were isolated and homogenized to extract total protein. Mouse IL-19 expression in kidney, liver, and lung were analyzed using direct ELISA. *P<0.05 compared with sham-operated mice. (C–E) Experimental mice were killed 2 days after renal IRI. RTQ-PCR was done with primers specific for IL-19, IL-20R1, and IL-20R2. The relative quantification of PCR products was expressed as 2^−ΔΔCt^, corrected using GAPDH expression, and relative to levels of untreated cells. Data are the means ± SD of three experiments. *P<0.05 compared with sham-operated mice.

AKI is frequently associated with distant organ dysfunction, especially the liver and lung [6], [7]. We hypothesized that IL-19 is not only involved in renal injury but also in other AKI injuries to vital organs; thus, we studied the tissue expression of IL-19 and its receptors in kidneys, livers, and lungs during AKI. We collected these three vital organs from IRI and control mice, and used IHC staining to detect the expression of IL-19 and its receptors. Both IL-19 and its receptors were upregulated in the kidneys, lungs, and livers of IRI mice (Figure 2A). ELISA showed that IL-19 levels were higher in the kidneys, lungs, and livers of IRI mice than the sham control mice (Figure 2B). RTQ-PCR also confirmed that transcripts of IL-19 and its receptors were higher in the liver and lung tissues of IRI mice than the sham control mice (Figure 2C–E). We also performed the IHC staining in the HgCl_2_-induced AKI rats and got the similar results (Figure S1 in File S1). These results suggested that IL-19 and its receptors were induced not only in renal tissue, but also in liver and lung tissue during the course of AKI.

### IL-19 upregulated TGF-β1, MCP-1, and IL-19 transcripts in mouse epithelial (M-1) cells

**Figure 3:**
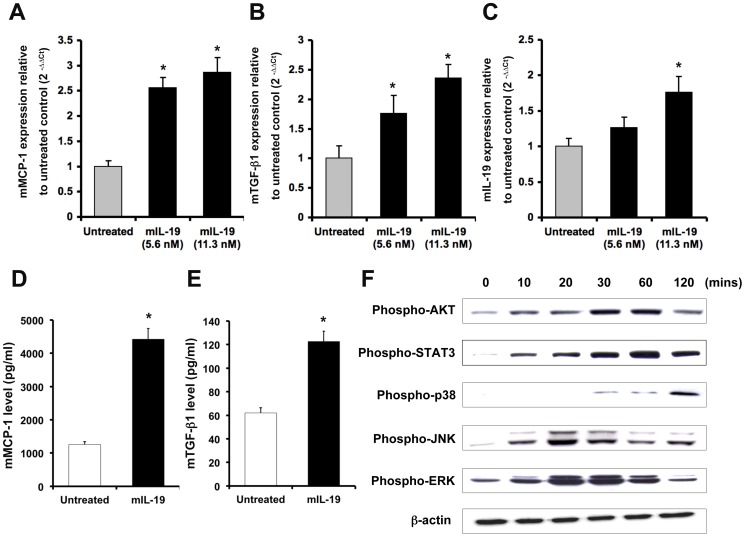
Functions of IL-19 in M-1 cells. (A–C) M-1 cells were treated with or without 11.3 nM (200 ng/ml) of mIL-19 for 6 h. RTQ-PCR was done with primers specific for MCP-1, TGF-β1, and IL-19. The relative quantification of PCR products was expressed as 2^−ΔΔCt^, corrected using GAPDH expression, and relative to levels of untreated cells. Data are the means ± SD of three experiments. *P<0.05 compared with untreated controls. (D–E) M-1 cells were seeded and starved for 10 h, and then stimulated with 11.3 nM (200 ng/ml) of IL-19 for 96 h. MCP-1 and TGF-β1 levels were measured by using specific ELISA kits. *P<0.05 compared with untreated controls. (F) M-1 cells were treated with 5.6 nM (100 ng/ml) mIL-19 for the indicated times. Cell lysates were collected and the levels of phospho-AKT, STAT3, p38 MAPK, JNK, and ERK 1/2 were detected using Western blotting with specific antibodies. β-actin was a loading control. Data are representative of 3 independent experiments.

AKI is an inflammatory disease of the kidney [16], [17]. Injured tubular epithelial cells generate a number of mediators that potentiate the inflammatory response, such as proinflammatory cytokines (TGF-β1, TNF-α, IL-6, and IL-1β) and chemotactic cytokines (MCP-1, CXCL10, and RANTES) [4], [18]. Exploring cytokine expression in the kidney during the inflammatory process may help clarify the pathophysiological mechanisms of AKI. To study the direct effects of IL-19 on renal tubular cells, we used a mouse epithelial cell line (M-1 cells) derived from cortical collecting ducts for the *in vitro* study. M-1 cells express IL-19 and its receptors (Figure S2 in File S1), and it was reported that IL-19 induces its own expression (auto-induction) in human peripheral blood mononuclear cells [19]. We therefore hypothesized that IL-19 produces IL-19 and other proinflammatory cytokines and chemokines in autocrine and paracrine manners in various organs. To test this possibility, we analyzed the effects of IL-19 on the production of cytokines and chemokines in M-1 cells. RTQ-PCR showed that MCP-1, TGF-β1, and IL-19 transcripts were upregulated after IL-19 treatment (Figure 3A–C). ELISA also confirmed that IL-19 induces MCP-1 and TGF-β1 production in M-1 cells (Figure 3D–E).

### IL-19 activated AKT, STAT3, p38 MAPK, JNK, and ERK 1/2 in M-1 cells

To evaluate the downstream signals induced by IL-19 in renal epithelial cells, M-1 cells were treated with IL-19, and cell lysates were analyzed using Western blotting with antibodies specifically against phosphorylated AKT, STAT3, p38 mitogen-activated protein kinase (MAPK), JNK, and ERK 1/2, which are associated with cell survival, cytokine expression, and apoptosis. The phosphorylation of AKT, STAT3, p38 MAPK, JNK, and ERK 1/2 was higher in IL-19-treated M-1 cells (Figure 3F).

### IL-19 dose-dependently and specifically induced cell death in M-1 cells

**Figure 4:**
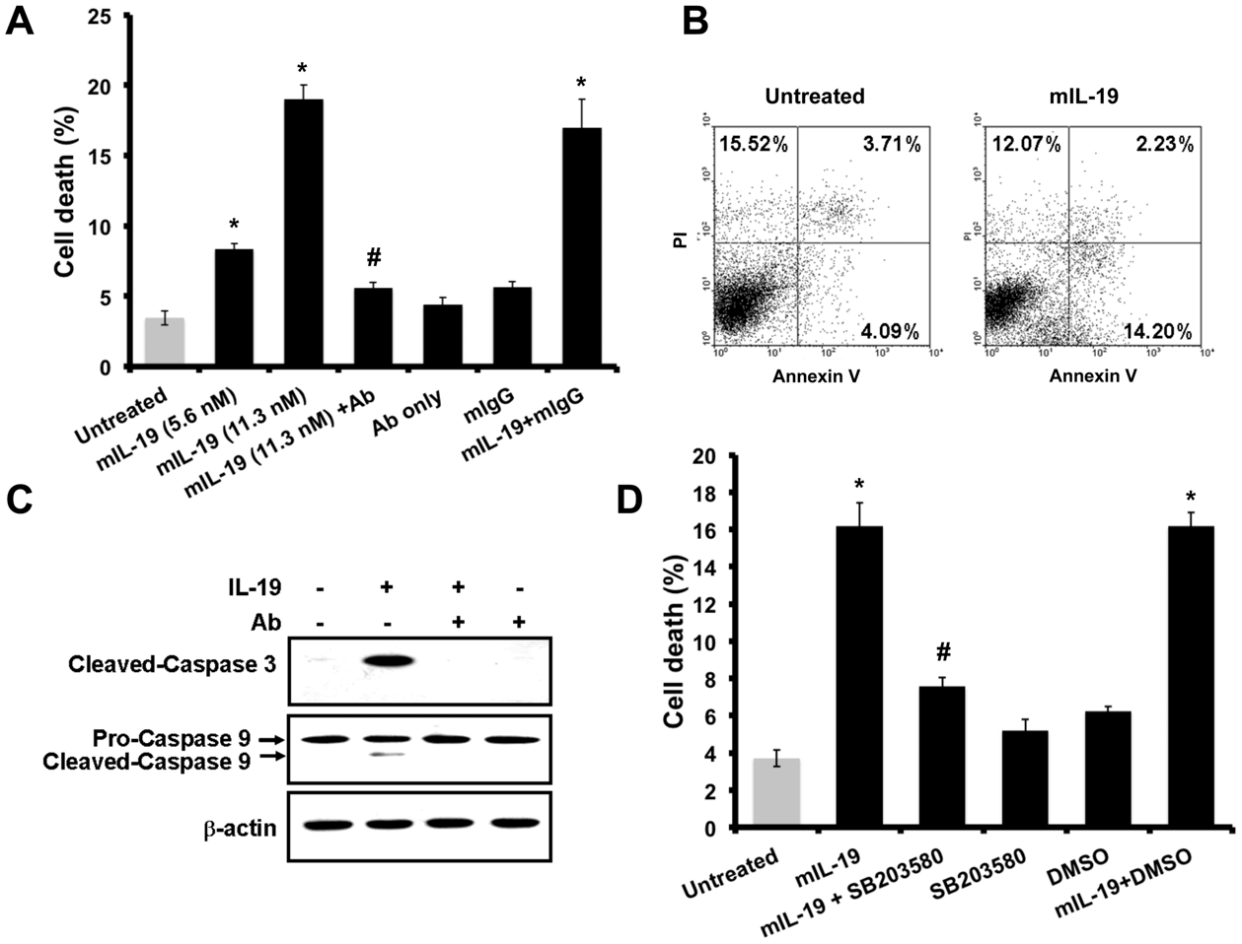
IL-19 induced cell apoptosis in M-1 cells. (A) Flow cytometric analysis of cell apoptosis in M-1 cells treated with mIL-19 (5.6 nM) (100 ng/ml), mIL-19 (11.3 nM) (200 ng/ml), Ab (anti-IL-20R1 mAb) (2 µg/ml), mIL-19 (11.3 nM) plus Ab (2 µg/ml), mIgG (isotype control Ab) (2 µg/ml), mIL-19 (11.3 nM) plus mIgG (2 µg/ml) for 24 h. The cells were fixed using ethanol, stained using propidium iodide (PI), and analyzed using flow cytometry. The percentages of dead cells (M1 region) are shown as a bar. Data are means ± SD of three experiments. *P<0.05 compared with untreated controls. ^#^ *P*<0.05 versus treatment with mIL-19 (11.3 nM). (B) M-1 cells were treated with or without mIL-19 for 17 h. The cells were stained with PI and annexin-V and then analyzed using flow cytometry. (C) M-1 cells were treated with mIL-19 (11.3 nM) (200 ng/ml), Ab (anti-IL-20R1 mAb) (2 µg/ml), or mIL-19 (11.3 nM) (200 ng/ml) plus Ab (2 µg/ml) for 24 h. Cell lysates were analyzed using Western blotting with specific antibodies. Cleaved-caspase 3 with a molecular weight of 19 kDa is shown. Pro- and cleaved-caspase 9 with molecular weights of 51 KDa and 39 kDa are shown. β-actin was an input control. (D) M-1 cells were treated with different reagents (11.3 nM (200 ng/ml) of mIL-19, 10 µM of SB203580, DMSO, mIL-19 plus SB203580, or mIL-19 plus DMSO) for 24 h. The cells were fixed using ethanol, stained using PI, and analyzed using flow cytometry. The percentages of dead cells (M1 region) are shown as a bar. Data are the means ± SD of three experiments. *P<0.05 compared with untreated controls. ^#^ *P*<0.05 versus treatment with mIL-19.

AKI leads to apoptosis and necrosis of renal tubular epithelial cells [2]. To determine whether IL-19 participated in the pathogenesis of AKI by inducing apoptosis of renal tubular epithelial cells, we treated M-1 cells with different concentrations of IL-19 for 24 h and then used flow cytometry to analyze the percentages of cell death. The result showed that IL-19 dose-dependently induced cell death in M-1 cells. In addition, anti-mIL-20R1 antibody against IL-19 receptor subunits IL-20R1 completely blocked IL-19-induced apoptosis in M-1 cells (Figure 4A).

### IL-19 induced mitochondria-dependent apoptosis by activating caspase 3 and 9 in M-1 cells

To investigate the pathway through which IL-19 induces cell death, we treated M-1 cells with or without IL-19 for 17 h, stained them with annexin-V/propidium iodide (PI), and then analyzed the percentage of apoptotic cells. IL-19 induced 14.2% apoptosis in M-1 cells (Figure 4B). There are two major apoptotic pathways in AKI: intrinsic and extrinsic. We used Western blotting with anti-caspase-3, -8, and -9 antibodies on the M1 cell lysate after IL-19 treatment. More cleaved caspase 3 and 9 was detected in IL-19-treated M-1 cells than in untreated cells. In addition, anti-mIL-20R1 antibody completely blocked the IL-19-induced cleavage of caspase 3 and 9 in M-1 cells (Figure 4C). Furthermore, IL-19 did not affect the level of caspase 8 (data not shown). These results indicated that mL-19 specifically induced apoptosis in M-1 cells by activating caspase 9, the mitochondrial pathway, and that anti-mIL-20R1 antibody neutralized the apoptotic effect of IL-19.

### IL-19 induced cell death through the p38 MAPK pathway in M-1 cells

IL-19 activated p38 MAPK in M-1 cells. It was reported [20] that okadaic acid-induced renal epithelial cell apoptosis was accompanied by the activation of the p38 MAPK pathway. Therefore, we investigated whether p38 MAPK also mediates IL-19-induced renal epithelial cell apoptosis. The p38 MAPK inhibitor SB203580 partially inhibited IL-19-induced cell death (Figure 4D), which indicated that the p38 MAPK was also involved in the IL-19-induced apoptosis pathway. The inhibitors of ERK or JNK did not reduce IL-19-induced apoptosis (data not shown).

### IL-19 upregulated TNF-α and IL-10 transcripts in HepG2 cells

**Figure 5:**
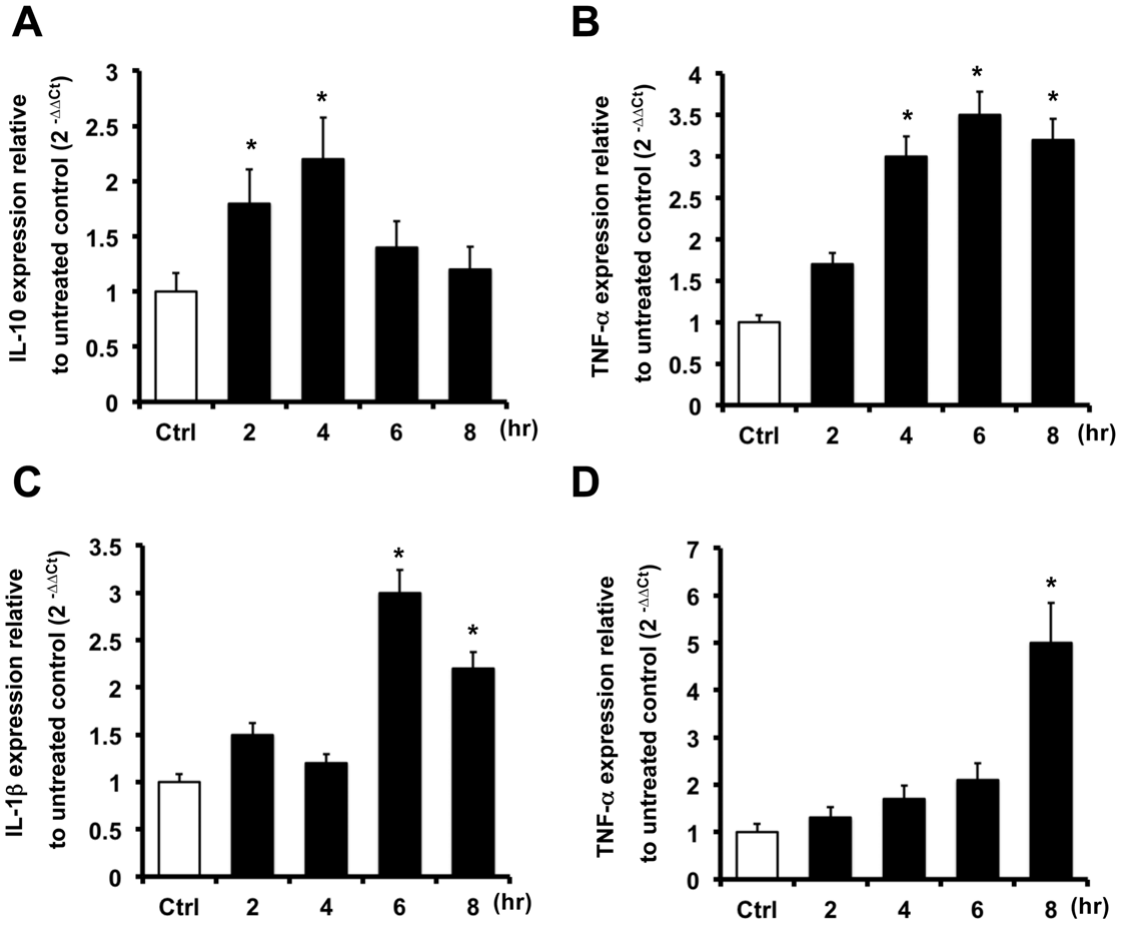
Functions of IL-19 in HepG2 and A549 cells. The cells were treated with hIL-19 (200 ng/ml) for the indicated times. RTQ-PCR was done with primers specific for IL-10, TNF-α, and IL-1β. The relative quantification of PCR products was expressed as 2^−ΔΔCt^, corrected using GAPDH expression, and relative to levels of untreated cells. Data are the means ± SD of three experiments. *P<0.05 compared with untreated controls.

AKI may cause hepatic damage, and hepatic levels of TNF-α and IL-10 increased significantly after renal IRI in mice [21], [22]. We observed that IL-19 was expressed *in vivo* in the liver of IRI mice. To determine whether IL-19 promotes the expression of TNF-α and IL-10, we analyzed the effect of IL-19 on the HepG2 human hepatoma cell line. RTQ-PCR showed that TNF-α and IL-10 transcripts were upregulated after IL-19 treatment (Figure 5A, B).

### IL-19 upregulated IL-1β and TNF-α transcripts in A549 cells

Clinical studies demonstrate that pulmonary complication is the one of the major contributors of mortality in AKI patients. AKI may affect the lungs via increased renal production or impaired clearance of mediators of lung injury, such as proinflammatory cytokines [8]. IL-1β and TNF-α mediate the development of numerous inflammatory lung diseases [23]. *In vivo*, we observed that IL-19 and its receptors were overexpressed in the lung epithelial cells of IRI mice. Therefore, we hypothesized that IL-19 might affect the lung function by regulating IL-1β and TNF-α expression. RTQ-PCR showed that IL-1β and TNF-α transcripts were upregulated in IL-19-treated A549 lung cells. These data confirmed our hypothesis (Figure 5C–D).

### IL-20R1 deficiency reduced the severity of renal failure in ischemic AKI mice

**Figure 6:**
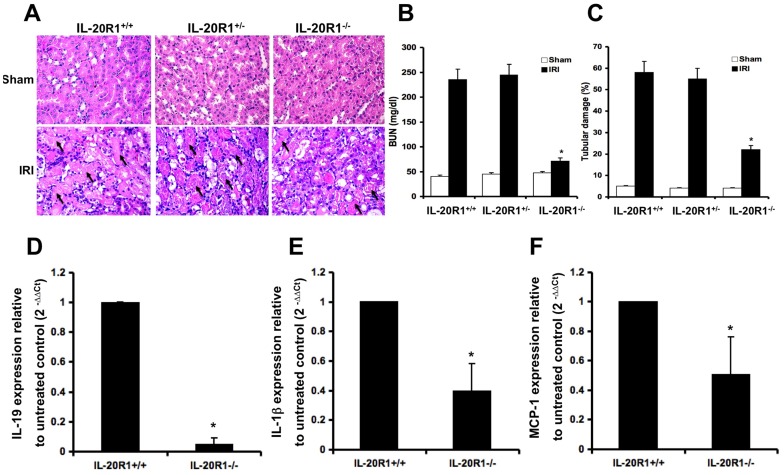
An IL-20R1 deficiency reduced the severity of IRI-induced AKI. (A) IL-20R1^+/+^ (*n* = 5), IL-20R1^+/−^ (*n* = 5), and IL-20R1^−/−^ (*n* = 5) mice were killed 4 days after renal IRI. Kidney sections from IL-20R1^+/+^, IL-20R1^+/−^, and IL-20R1^−/−^ mice were stained with hematoxylin and eosin (magnification: ×400). Arrows indicate the damaged tubular cells. (B) Serum BUN levels of AKI-IL-20R1^+/+^ (*n* = 5), AKI-IL-20R1^+/−^ (*n* = 5), and AKI-IL-20R1^−/−^ (*n* = 5) mice were analyzed on day 4. Data are the means ± SD of three experiment. *P<0.01 compared with AKI-IL-20R1^+/+^ mice. (C) Quantitative analysis of the area of damaged tubular cells from IL-20R1^+/+^ (*n* = 5), IL-20R1^+/−^ (*n* = 5), and IL-20R1^−/−^ (*n* = 5) mice 4 days after renal IRI. Data are the means ± SD of three experiments. *P<0.05 compared with AKI-IL-20R1^+/+^ mice. (D–E) The expression of IL-19, IL-1β, and MCP-1 in AKI-IL-20R1^+/+^ (*n* = 5) and AKI-IL-20R1^−/−^ (*n* = 5) mice was analyzed on day 4. RTQ-PCR was done with primers specific for IL-19, IL-1β, and MCP-1. The relative quantification of PCR products was expressed as 2^−ΔΔCt^, corrected using GAPDH expression, and relative to levels of untreated cells. Data are the means ± SD of three experiments. *P<0.05 compared with AKI-IL-20R1^+/+^ mice.

IL-19 is critical in the pathogenesis of AKI. IL-19 binds to a heterodimer receptor complex, IL-20R1/IL-20R2. Anti-mIL-20R1 antibody completely blocked the IL-19-induced cleavage of caspase 3 and 9 in M-1 cells (Figure 4C), which suggested that IL-20R1 is involved in IL-19-mediated apoptosis. Therefore, we investigated the effect of IL-20R1 deficiency on IL-19-mediated renal dysfunction. We successfully established a strain of IL-20R1 deficiency mice [24]. We used IRI on these IL-20R1 deficiency mice and assessed the severity of their renal failure. H&E staining showed that the damage to kidney tissue was lower in AKI-IL-20R1^−/−^ mice than in AKI-IL-20R1^+/+^ mice (Figure 6A), which indicated that the IL-20R1 deficiency protected against kidney damage in AKI mice. In addition, serum BUN levels were significantly lower in AKI-IL-20R1^−/−^ mice than in AKI-IL-20R1^+/+^ mice (*P*<0.05) (Figure 6B). Tubular damage during AKI is an indication of renal dysfunction. Thus, we measured the average tubular damage area of the cross-section of kidney tissue. The average tubular damage area was lower in AKI-IL-20R1^−/−^ mice than in AKI-IL-20R1^+/+^ mice (Figure 6C). We also found a similar protective effect in the HgCl_2_-induced AKI mouse model (Figure S3 in File S1). Furthermore, RTQ-PCR showed that IL-19, IL-1β and MCP-1 transcripts were downregulated in AKI-IL-20R1^−/−^ mice compared with AKI-IL-20R1^+/+^ mice (Figure 6D–E). Moreover, ELISA showed that IL-19 and TNF-α level in the liver and kidney tissue was lower in AKI-IL-20R1^−/−^ mice than in AKI-IL-20R1^+/+^ mice. These data provided evidence that IL-19 induced inflammatory response in distal organ during AKI by regulating TNF-α expression (Figure S4 in File S1). These results demonstrated that the IL-20R1-deficiency improved renal function and reduced the severity of renal damage in mice with IRI-induced AKI.

## Discussion

IL-19 is involved in many diseases, for example, psoriasis [25], asthma [15], and endotoxic shock [14]. The present study demonstrated that the protein and mRNA levels of IL-19 and its receptors were higher in the kidney, liver, and lung tissue in rodents with AKI than in the sham controls. *In vitro* studies showed IL-19 effects on cultured renal tubular, liver, and lung cells; furthermore, the renal injury in IL-19 receptor subunit IL-20R1 deficient mice attenuated when compared with the control mice with IRI- and HgCl_2_-induced AKI. This is the first study which shows that IL-19 may mediate the renal injury of both IRI and nephrotoxic AKI models, and that IL-19 is also associated with hepatic and pulmonary injuries in AKI.

IL-19 upregulated TGF-β1, MCP-1, and IL-19 transcript in renal epithelial M1 cells *in vitro*. TGF-β1 may promote the apoptosis of tubular cells by activating caspases [26], and inducing an epithelial-to-mesenchymal transition or α-smooth muscle actin expression in the renal cells [27], which may contribute to interstitial fibrosis through the excessive deposition of extracellular matrix. Therefore, we speculated that IL-19 may induces cell apoptosis through regulating TGF-β1 expression. MCP-1 is a chemoattractant that induces the inflammatory activation of tubular epithelial cells and contributes to tubulointerstitial inflammation. IL-19 upregulated MCP-1 and amplified the inflammatory response by recruiting monocytes to injury sites in the kidneys. IL-19 induced IL-19 expression in M-1 cells, and it may have formed a positive loop that amplified its effects on renal injury. We previously [12] showed that IL-19 induced IL-6, TNF-α, and apoptosis in mouse monocytes. Therefore, monocytes may also contribute to amplifying the inflammatory response in injured kidney tissue as well as in renal epithelial cells. These findings are supported by the *in vivo* data in the present study, which showed that the mRNA and protein levels of IL-19 increased in the kidney tissue of mice with IRI- and HgCl_2_-induced AKI.

IL-19 also increased the phosphorylation of AKT, STAT3, and MAP kinases. In the progression of AKI, the phosphorylation of AKT is critical for controlling the balance between survival and apoptosis [28]. The STAT3 signal is involved in the synthesis of TGF-β1, TIMP-1, and ECM protein [29], [30], which would increase the loss of functional renal tissue, and interstitial fibrosis. Therefore, IL-19 activation on AKT and STAT3 in M-1 cells supports the notion that IL-19 induces renal dysfunction. ERK 1/2 and JNK activation is required for apoptosis in renal epithelial cells [31]–[33]. In addition, p38 MAPK signaling mediates mechanical stretch-induced apoptosis in renal epithelial cells [34]. In the present study, IL-19 activated ERK 1/2, JNK, and p38 MAPK in M-1 cells; it also blocked p38 MAPK signaling, which attenuated IL-19-induced apoptosis. These findings suggested that IL-19 induces apoptosis in renal epithelial cells through these three pathways.

The pathophysiological role of IL-19 in AKI was further supported by the IL-19 receptor IL-20R1 subunit knockout studies. IL-19 and its receptors were upregulated in the kidney tissue of IRI mice and rats with HgCl_2_-induced AKI. After renal IRI or a nephrotoxic dose of HgCl_2_ treatment, IL-20R1-deficient mice showed lower levels of serum BUN and less tubular damage than did wild-type controls, which indicated that blocking the IL-19 signaling could improve kidney function *in vivo*. This protective effect may be attributable to the systemic or local absence of IL-19 signaling in IL-20R1-deficient mice. The result further provided evidence that IL-19 is an important mediator in both IRI and HgCl_2_-induced AKI. However, our data in HgCl_2_-induced AKI may have some discrepancy with other finding which showed HgCl_2_-induced AKI is independent of inflammation [35], [36].

AKI induces oxidative stress and promotes inflammation, apoptosis, and tissue damage in hepatocytes [21]. In addition, AKI may lead to the dysregulation of salt and water channels in lung tissue, which may modulate lung dysfunction, susceptibility to lung injury, or both [37]. However, the pathophysiological mechanism of the distant organ dysfunction in AKI is still undefined. In our *in vivo* studies, the hepatic and pulmonary mRNA and protein levels of IL-19 and its receptors increased in both IRI and HgCl_2_-induced AKI. In the subsequent *in vitro* studies, IL-19 raised the level of TNF-α and IL-10 transcripts in hepatoma cells, which may cause liver inflammation and lead to hepatic damage. IL-19 upregulated IL-1β and TNF-α in lung epithelial cells, which may promote an inflammatory response and cause pulmonary injury [8], [38]. Taken together, our data suggest that IL-19 generated from the injured kidney, from a distant organ, or systemically may be critical in distant organ dysfunction in AKI. However, unlike M1 cells, IL-19 did not induce IL-19 transcripts in hepatoma cells and lung epithelial cells, which suggests that there is no auto-induction in the liver or lungs.

Our *in vitro* experiments showed that epithelial cell was a target for IL-19. However, the expression of IL-19 and its receptors on other cells in the kidney, namely renal fibroblasts and mesangial cells, may also contribute differently to kidney diseases. We previously [39], [40] showed that IL-20 also targeted renal epithelial cells and induced cell apoptosis in AKI. Therefore, attenuation of kidney injury in the AKI model of IL-20R1-deficiency mice might be attributed to both 19 and IL-20 because these two cytokines share the same receptor complexes IL-20R1/IL-20R2. Therefore, further studies investigating the detailed molecular mechanism that regulates IL-19 and IL-20, and the crosstalk between these two cytokines in these three cell types will be clinically important, especially the question of whether there is an additive or synergistic effect between IL-19 and IL-20 in the course of AKI. Moreover, we found that IL-19 mediated the renal injury in both IRI and nephrotoxic AKI mice models. Whether IL-19 also plays critical role and has clinical relevance in the human AKI awaits further investigation.

Histologic examination demonstrated cellular damage in the outer and inner medullary-collecting duct, as well as in the proximal tubule in bilateral renal IRI-induced and HgCl_2_-induced AKI animal models. Therefore, we chose collecting duct cell line as a target cell to clarify the role of IL-19 in the pathogenesis of AKI. Whether IL-19 also acts on proximal tubule during AKI awaits future investigation.

IL-19 acts as a proinflammatory factor in psoriasis, asthma, sepsis, and RA [14], [15], [25], [41]. Our present study demonstrated that IL-19 acts as a proinflammatory cytokine in AKI. To the contrary, however, in inflammatory bowel disease, endogenous IL-19 appears to be protective. In experimental dextran sulfate sodium-induced acute colitis, IL-19^−/−^ mice were more susceptible to colitis than were wild-type controls [42]. A recent study [43] showed that IL-19 inhibits vascular smooth muscle cell motility by activating cytoskeletal and migratory regulatory proteins. These data imply that IL-19 may function as a dual-edged sword, depending on the cell types and disease entities.

In conclusion, our study demonstrates that IL-19 is an inflammatory mediator involved in AKI. IL-19 and its receptors were upregulated in the kidneys of ischemic AKI mice and rats with HgCl_2_-induced AKI. *In vitro*, IL-19 induced the expression of itself (auto-induction) and other proinflammatory cytokines, and activated the cell apoptosis process. IL-19 acted on hepatocytes and promoted the expression of TNF-α and IL-10, and also upregulated IL-1β and TNF-α expression in lung epithelial cells. Most importantly, we demonstrated that the IL-20R1-deficiency attenuated kidney injury in IRI and HgCl_2_-induced AKI. Collectively, our findings provide evidence that IL-19 targets several major organs and is involved in the pathogenesis of AKI. Thus, IL-19 or its receptor may be novel targets for treating renal injury after AKI. Potential new drugs for renal injury include antibodies or small molecules that neutralize IL-19 or block the cell-surface receptors of IL-19.

## Supporting Information

File S1 **Supporting information figures.** Figure S1. Expression of IL-19 and its receptors in three vital organs of rats with HgCl_2_-induced AKI. AKI rats (*n* = 3 in each group) were killed 48 h after they had been injected with HgCl_2_. Paraffined sections of kidney, liver, lung tissue were stained using anti-IL-19, -IL-20R1, and -IL-20R2 monoclonal antibodies. Anti-mIgG_1_ was a negative control. The reaction was detected using AEC chromogen stain (red), and the nuclei were counterstained with hematoxylin (blue). The bars represent 50 µm. All three rats in each group showed similar patterns. Shown sections are representative of three individual rats. Figure S2. Expression of IL-19 and its receptors in M-1 cells. The mRNA of M-1 cells was isolated for RT-PCR analysis using IL-19-, IL-20R1-, and IL-20R2- specific primers. GAPDH was the internal control. Neg indicates non-template negative control. All experiments were done 3 times with similar results. Data are from a representative experiment. Figure S3. An IL-20R1 deficiency reduced the severity in mice with HgCl_2_-induced AKI. (A) IL-20R1^+/+^ (*n* = 5), IL-20R1^+/−^ (*n* = 5), and IL-20R1^−/−^ (*n* = 5) mice were killed 4 days after they had been injected with HgCl_2_. Kidney sections from IL-20R1^+/+^, IL-20R1^+/−^, and IL-20R1^−/−^ mice were stained with hematoxylin and eosin (magnification: ×400). Arrows indicate the damaged tubular cells. (B) Serum BUN levels of AKI-IL-20R1^+/+^ (*n* = 5), AKI-IL-20R1^+/−^ (*n* = 5), and AKI-IL-20R1^−/−^ (*n* = 5) mice were analyzed on day 3. Data are the means ± SD of three experiments. *P<0.01 compared with AKI-IL-20R1^+/+^ mice. (C) Quantitative analysis of the area of damaged tubular cells from IL-20R1^+/+^ (*n* = 5), IL-20R1^+/−^ (*n* = 5), and IL-20R1^−/−^ (*n* = 5) mice 4 days after they had been injected with HgCl_2_. Data are the means ± SD of three experiments. * *P*<0.05 compared with AKI-IL-20R1^+/+^ mice. Figure S4. An IL-20R1 deficiency reduced IL-19 and TNF-α production in IRI-induced AKI. IL-20R1^+/+^ (*n* = 5) and IL-20R1^−/−^ (*n* = 5) mice were killed 4 days after renal IRI. Liver and lung tissues from IL-20R1^+/+^ and IL-20R1^−/−^ mice were homogenized with PBS, then centrifuged, and collect supernatant to analyze IL-19 and TNF-α level by using ELISA. Data are the means ± SD of three experiments. *P<0.05 compared with AKI-IL-20R1^+/+^ mice.
(PDF)

Table S1 Primers used for gene expression analysis in the study.
(DOC)
