# Supplementary figures and images for: Interleukin-19 Mediates Tissue Damage in Murine Ischemic Acute Kidney Injury
Source: PLoS One. 2013 Feb 26;8(2):e56028. doi: 10.1371/journal.pone.0056028 (PMC3582636; doi:10.1371/journal.pone.0056028)

**Supplementary Figure**

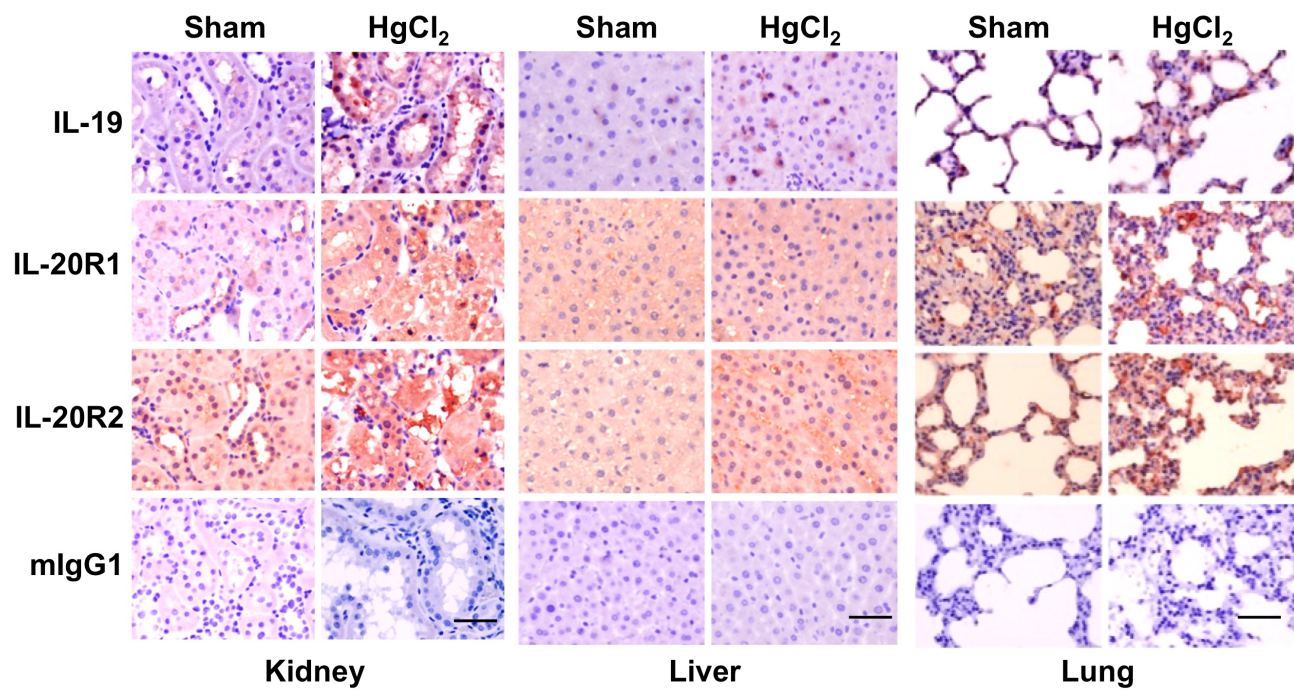

**Figure S1**

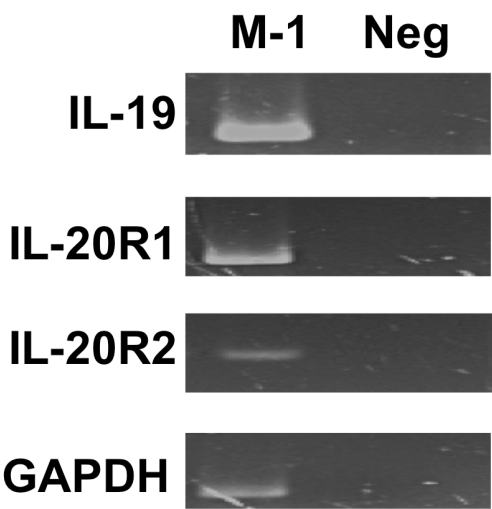

**Figure S2**

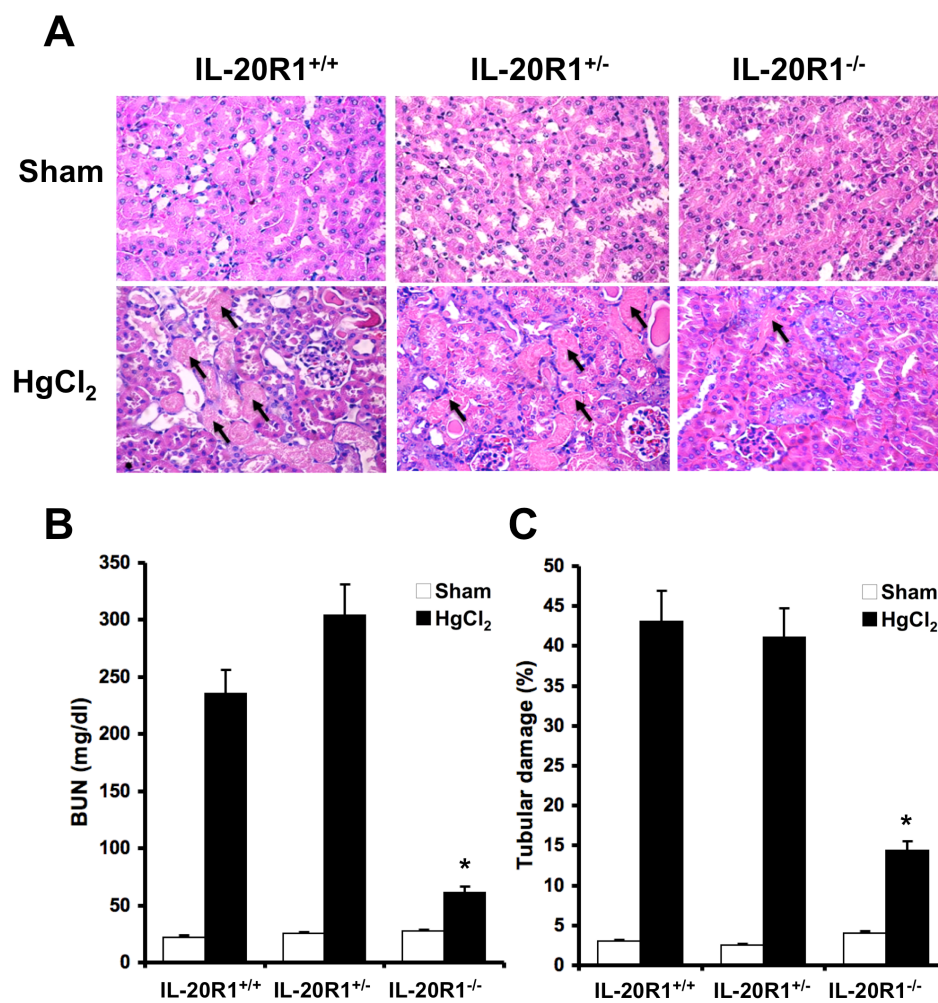

Figure S3

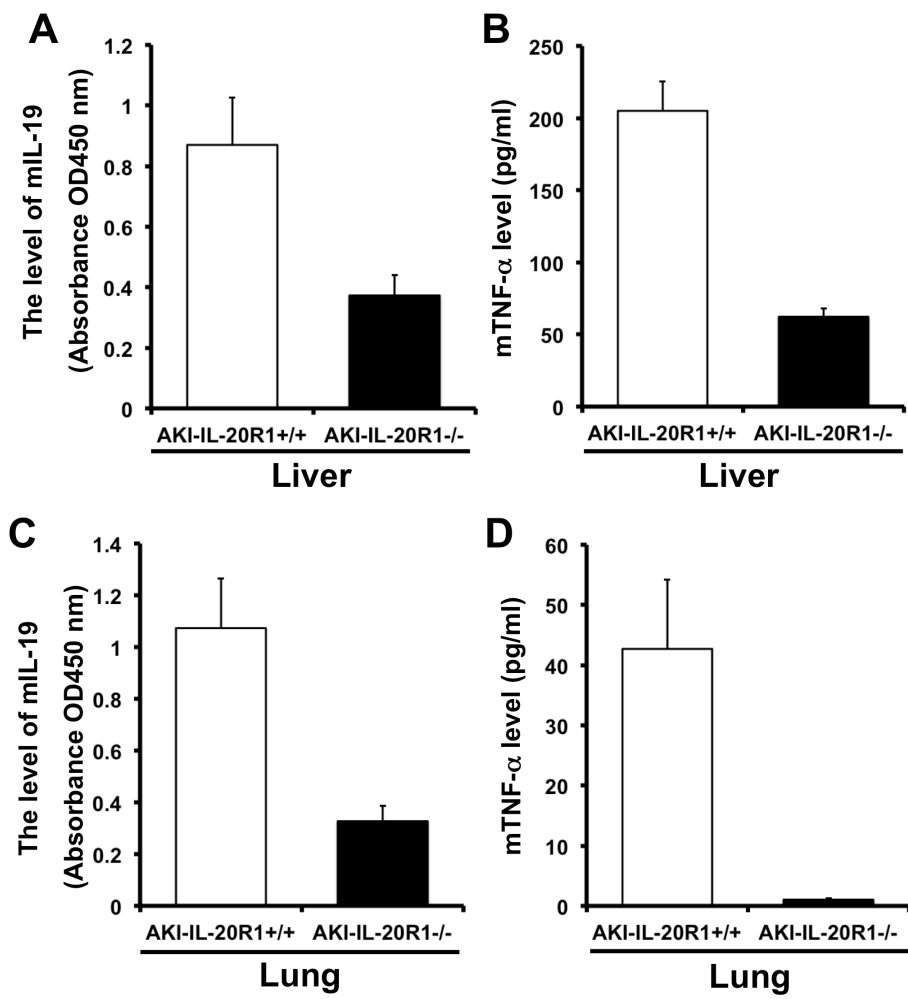

Figure S4

Supplement: File S1 — Supporting information figures. Figure S1. Expression of IL-19 and its receptors in three vital organs of rats with HgCl2-induced AKI. AKI rats (n = 3 in each group) were killed 48 h after they had been injected with HgCl2. Paraffined sections of kidney, liver, lung tissue were stained using anti-IL-19, -IL-20R1, and -IL-20R2 monoclonal antibodies. Anti-mIgG1 was a negative control. The reaction was detected using AEC chromogen stain (red), and the nuclei were counterstained with hematoxylin (blue). The bars represent 50 µm. All three rats in each group showed similar patterns. Shown sections are representative of three individual rats. Figure S2. Expression of IL-19 and its receptors in M-1 cells. The mRNA of M-1 cells was isolated for RT-PCR analysis using IL-19-, IL-20R1-, and IL-20R2- specific primers. GAPDH was the internal control. Neg indicates non-template negative control. All experiments were done 3 times with similar results. Data are from a representative experiment. Figure S3. An IL-20R1 deficiency reduced the severity in mice with HgCl2-induced AKI. (A) IL-20R1+/+ (n = 5), IL-20R1+/− (n = 5), and IL-20R1−/− (n = 5) mice were killed 4 days after they had been injected with HgCl2. Kidney sections from IL-20R1+/+, IL-20R1+/−, and IL-20R1−/− mice were stained with hematoxylin and eosin (magnification: ×400). Arrows indicate the damaged tubular cells. (B) Serum BUN levels of AKI-IL-20R1+/+ (n = 5), AKI-IL-20R1+/− (n = 5), and AKI-IL-20R1−/− (n = 5) mice were analyzed on day 3. Data are the means ± SD of three experiments. *P<0.01 compared with AKI-IL-20R1+/+ mice. (C) Quantitative analysis of the area of damaged tubular cells from IL-20R1+/+ (n = 5), IL-20R1+/− (n = 5), and IL-20R1−/− (n = 5) mice 4 days after they had been injected with HgCl2. Data are the means ± SD of three experiments. * P<0.05 compared with AKI-IL-20R1+/+ mice. Figure S4. An IL-20R1 deficiency reduced IL-19 and TNF-α production in IRI-induced AKI. IL-20R1+/+ (n = 5) and IL-20R1−/− (n = [file pone.0056028.s001.pdf]
