# Supplementary material for: Interleukin-19 Mediates Tissue Damage in Murine Ischemic Acute Kidney Injury
Source: PLoS One. 2013 Feb 26;8(2):e56028. doi: 10.1371/journal.pone.0056028 (PMC3582636; doi:10.1371/journal.pone.0056028)
Supplement: Table S1 — Primers used for gene expression analysis in the study. (DOC) [file pone.0056028.s002.doc]

**Primer sequence (5’-3’)**

**Gene Forward Reverse**

**mIL-19** TGGAGAACCTCAGGAGCATT GAATGTCAGCAGGTTGTTGG

**mIL-20R1** CTAAGTCGAGAAGAACGTGGT TGACTTTAGCCTTCCATGCTGA

**mIL-20R2** GTGCACCTAGAAACCATGGA CCATCTTCCAGACGGAGAG

**mTGF-β1**  ATTCAGCGCTCACTGCTCTT CTTCTCTGTGGAGCTGAAGCA

**mIL-1β** GCTGAAAGCTCTCCACCTCA CTTGGGATCCACACTCTCCAG

**mGAPDH** AGCCTCGTCCCGTAGACAAAA GATGACAAGCTTCCCATCTCG

**hIL-10** GGCGCTGTCATCGATTTCTTC GCCACCCTGATGTCTCAGTT

**hIL-1β** TACCTGTCCTGCGTGTTGAA TCTTTGGGTAATTTTTGGGATCT

**hTNF-α** TGCTTGTTCCTCAGCCTCTTCT TATCTCTCAGCTCCACGCCATT

m indicates mouse; h, human.
